# Telomerase-targeting compounds Imetelstat and 6-thio-dG act synergistically with chemotherapy in high-risk neuroblastoma models

**DOI:** 10.1007/s13402-022-00702-8

**Published:** 2022-08-12

**Authors:** Janina Fischer-Mertens, Felix Otte, Andrea Roderwieser, Carolina Rosswog, Yvonne Kahlert, Lisa Werr, Anna-Maria Hellmann, Maya Berding, Bill Chiu, Christoph Bartenhagen, Matthias Fischer

**Affiliations:** 1 Experimental Pediatric Oncology, University Children’s Hospital of Cologne, Medical Faculty, University of Cologne, Cologne, Germany; 2 Center for Molecular Medicine Cologne (CMMC), University of Cologne, Cologne, Germany; 3 Department of Internal Medicine I, University Hospital of Cologne, Cologne, Germany; 4 Pediatric Surgery, Stanford University, Stanford, CA USA

**Keywords:** Neuroblastoma, Drug testing, Pediatric cancer, Animal models

## Abstract

**Background:**

The majority of high-risk neuroblastomas harbor telomerase activity, and telomerase-interacting compounds, such as 6-thio-2’-deoxyguanosine (6-thio-dG), have been found to impair the growth of telomerase-positive neuroblastoma cell lines. It has remained unclear, however, how such drugs can be combined with other compounds used in current treatment concepts for neuroblastoma patients.

**Methods:**

Growth-inhibitory effects of varying concentrations of 6-thio-dG in combination with etoposide, doxorubicin or ceritinib were determined in eight telomerase-positive neuroblastoma cell lines with distinct genetic backgrounds. Tumor growth inhibition of subcutaneous xenografts from three different cell lines was assessed upon treatment with 6-thio-dG, the competitive telomerase inhibitor imetelstat, etoposide, or combinations of these compounds.

**Results:**

Robust synergistic anti-tumor effects were observed for combinations of 6-thio-dG and etoposide or doxorubicin, but not for 6-thio-dG and ceritinib, in telomerase-positive neuroblastoma cell lines *in vitro*. Treatment of mouse xenografts with combinations of 6-thio-dG and etoposide significantly attenuated tumor growth and improved mouse survival over etoposide alone in two of three cell line models. Treatment of xenograft tumors by imetelstat monotherapy decreased telomerase activity by roughly 50% and significantly improved survival over control in all three models, whereas treatment with imetelstat plus etoposide led to enhanced survival over etoposide monotherapy in one model. Mechanistically, the synergistic effect was found to be due to both increased apoptosis and cell cycle arrest.

**Conclusion:**

Our study indicates that telomerase is an actionable target in telomerase-positive neuroblastoma, and demonstrates that combination therapies including telomerase-interacting compounds may improve the efficacy of established cytotoxic drugs. Targeting telomerase may thus represent a therapeutic option in high-risk neuroblastoma patients.

**Supplementary Information:**

The online version contains supplementary material available at 10.1007/s13402-022-00702-8.

## Background

Neuroblastoma is a pediatric solid tumor of the sympathetic nervous system. Clinical courses of neuroblastoma patients vary greatly, ranging from spontaneous regression to fatal progression despite intensive multimodal treatment [1]. It has been demonstrated recently that the presence of telomere maintenance mechanisms is a hallmark of high-risk neuroblastoma [2]. Activation of telomere maintenance mechanisms is an essential prerequisite for cancer cells to gain immortal proliferation capacity [3], and may be conferred by either induction of the reverse transcriptase telomerase or by activation of the alternative lengthening of telomeres (ALT) pathway [4]. Most high-risk neuroblastomas exhibit high levels of telomerase activity, which are caused by induction of the telomerase reverse transcriptase (*TERT*) gene through *MYCN* over-expression in *MYCN*-amplified tumors, or through genomic rearrangements of the *TERT* locus [5, 6]. In another small fraction of high-risk tumors, *TERT* is upregulated by unknown mechanisms [4]. By contrast, telomere maintenance is conferred by ALT in roughly one-third of high-risk neuroblastomas, which is associated with inactivating mutations of the *ATRX* gene [2, 4, 5, 7]. In addition to telomere maintenance mechanisms, mutations in genes related to the *RAS* pathway, such as the anaplastic lymphoma kinase (*ALK*) gene, and the p53 pathway have been found to contribute to neuroblastoma pathogenesis and to be associated with a poor clinical outcome, in particular in the presence of telomere maintenance mechanisms [2, 8, 9].

Telomerase has been considered as a promising therapeutic target in cancer for many years, however, both the development of effective and specific telomerase inhibitors and incorporation of available telomerase inhibitors into clinical practice has remained challenging [4]. It has been demonstrated recently that the telomerase substrate nucleoside precursor 6-thio-2’-deoxyguanosine (6-thio-dG) significantly impairs growth of telomerase-positive tumors in preclinical models, including neuroblastoma cell lines harboring *TERT* rearrangements, *MYCN* amplification or high *TERT* expression in the absence of these alterations, both *in vitro* and *in vivo* [4, 10–13]. The nucleoside analogue 6-thio-dG is recognized by telomerase and incorporated into *de novo* synthesized telomeres, which results in modified telomeres, leading to telomere dysfunction without substantial changes of telomere lengths [10]. Another telomerase-interacting compound is imetelstat, a covalently-lipidated 13-mer thiophosphoramidate oligonucleotide, which binds with high affinity to the template region of the RNA component of human telomerase (*hTERC*) and potently and competitively inhibits telomerase enzymatic activity [14]. Clinical results from imetelstat phase 2 studies demonstrated clinical benefits including durable transfusion independence in lower risk myelodysplastic syndromes and improved overall survival in relapsed/refractory myelofibrosis [15, 16]. Imetelstat is currently in phase 3 clinical trials in these diseases. The therapeutic potential of imetelstat in high-risk neuroblastoma has not been evaluated yet. In addition, it is unclear how a telomerase-directed therapy could be integrated into existing treatment concepts for high-risk neuroblastoma. We therefore aimed to examine whether telomerase-interacting compounds in combination with established chemotherapy or *ALK* inhibitors act synergistically on neuroblastoma growth *in vitro* and *in vivo*.

## Material and methods

### Neuroblastoma cell lines

The NB cell-lines GI-MEN, CLB-GA, LS and IMR-32 were purchased from the German Collection of Microorganisms and Cell Cultures (DSMZ). The cell lines SK-N-FI, BE(2)-C, TR14 and SH-SY5Y were obtained from the American Tissue Culture Collection (ATCC). Cell lines were cultured at 37 °C and 5% CO_2_ and temporarily stored in DMSO at -80 °C. CLB-GA, GI-MEN, BE(2)-C, IMR-32, TR-14, LS and SH-SY5Y were maintained in RPMI-GlutaMAX™ with 10% fetal calf serum (FCS), while SK-N-FI was maintained in DMEM supplemented with 10% FCS and 1% non-essential amino acids (Thermo Fisher Scientific, Carlsbad, CA, USA). *TERT* expression was determined by oligonucleotide microarray analysis (Agilent Technologies, Santa Clara, CA, USA) for all cell lines according to manufacturer’s guidelines.

### Telomerase interacting compounds

6-thio-dG was purchased from BioVision (Milpitas, CA, USA), and imetelstat was kindly provided by Geron (Foster City, CA, USA).

### Analysis of drug synergy *in vitro*

Growth-inhibitory effects of varying concentrations of 6-thio-dG in combination with etoposide (VP-16, Selleckchem, USA), doxorubicin (Merck, Germany) or ceritinib (LDK378, Selleckchem, USA) on neuroblastoma cell lines with *TERT* rearrangements (CLB-GA, GI-ME-N), high *TERT* expression (SH-SY5Y) or *MYCN* amplification (BE(2)-C, IMR-32, Kelly, LS, TR-14) were determined after 96 h of substance incubation using CellTiter-Glo®. A semi-automated substance screening protocol was established on a Biomek 4000 Automated Workstation (Beckman Coulter, Brea, CA, USA). Single or combined compounds in nine different concentrations and DMSO as control were added in triplicates 24 h after the cells were seeded in a 96-well plate at a density of 3000 cells/well. Cell viability was measured after 96 h of substance incubation using a CellTiter-Glo® (CTG) Luminescent Cell Viability Assay (Promega, Madison, WI, USA). In brief, 100 µl CTG-reagent was added to the wells and incubated for 30 min at room temperature. Signals were measured using a SpectraMax® i3x Multi-Mode microplate reader (Molecular Devices, San Jose, CA, USA). Drug synergy was calculated according to the Chou-Talalay combination index method [17, 18].

### Western blotting

Neuroblastoma cells were seeded into 25 cm^2^ cell culture flasks and incubated for 48 h with varying drug concentrations after reaching medium density. Total protein was extracted using RIPA buffer (1% NP-40, 0.1% sodium dodecyl sulfate, 0.5% sodium deoxycholate) and protease inhibitors. Protein concentrations were quantified using the bicinchoninic acid bradford method. Protein lysates (50 μg) were separated using 10% SDS-PAGE4-12% Bis–Tris Gels and electrotransferred to polyvinylidene difluoride nitrocellulose membranes (Biosharp, USA). The membranes were blocked in PBST 5% non-fat dry milk and TBST solution, incubated in TBST and gently shaken with primary antibodies diluted to vendor specifications at 4 °C overnight. Finally, the membranes were incubated with appropriate secondary antibodies and visualized using an enhanced chemiluminescence detection kit (Alpha Innotech, USA) or chemiluminescence detection kit (Pierce ThermoFisher, USA). Antibodies directed against PCNA (rabbit), cleaved caspase-7 (rabbit), p21Waf1/Cip1 (rabbit), PARP (rabbit), anti-rabbit IgG HRP linked and β-Actin (rabbit) were purchased from Cell Signaling Technology (Frankfurt/Main, Germany), and against β-Actin (mouse) from Abcam (UK). An antibody directed against pCDK1 was purchased from Life Technologies (Darmstadt, Germany) and a polyclonal goat anti-mouse Ig/HRP from Agilent (Ratingen, Germany).

### Flow cytometry

Neuroblastoma cells were seeded in 60 mm cell culture dishes (Sarstedt, Germany) at a density of 0.1 × 10^5^ cells/ml. After two days the cells were treated with varying drug concentrations for 48 h. As a control, cells were treated with DMSO. Cells were harvested using accutase (Sigma-Aldrich). For cell cycle analysis cells and supernatants were harvested and washed twice with FACS buffer (PBS, 5% FCS, 2 mM EDTA) and filtered through a 40 µm cell strainer (Corning, USA). The pellet was subsequently resuspended in 500 µl PBS and permeabilized and fixed in ice-cold 70% ethanol overnight. After washing twice with PBS the cells were stained with PI after RNAse A was added. For apoptosis staining, cells and supernatants were harvested and washed twice with cell staining buffer (Biolegend, USA). As positive control, cells were incubated at 70 °C for 5 min. Cells were resuspended in Annexin Binding Buffer, PI and Annexin-Alexa 488 following the manufacturer’s instructions (Biolegend, USA), after which RNAse A was added. Cells were analyzed using a FACSCanto II instrument (BD Biosciences) and FlowJo software (BD Biosciences).

### Telomeric repeat amplification protocol assay (TRAP assay)

Telomerase activity in cell lines and neuroblastoma xenografts was determined using a PCR-based telomeric repeat amplification protocol (TRAP) enzyme-linked immunosorbent assay (ELISA) kit (TeloTAGGG Telomerase PCR ELISAPLUS, Roche) according to the manufacturer’s protocol. Briefly, the assay was performed in a 40 µl reaction mixture containing 20 mM Tris–HCl (pH 8.0), 1 mM dNTPs, and aliquots of proteins from T24 cell extracts (control), cell lines or tissue samples. Each mixture was incubated at 20 °C for 60 min. Telomerase activity was measured using SpectraMax at 450 nM. Tumors were excluded from the study when the extracted protein levels were too low for the TRAP assay, due to large areas of necrosis resulting in small residual tumor masses.

### Telomere length assay

DNA was isolated from tumor samples or cell lines using a Gentra Puregene Tissue Kit (Qiagen, Cat.No. 158667). Mean telomer restriction fragments (TRF) were determined using a Telo-TAGGG Telomere Length Assay kit (Roche, Cat. No. 12 209 136 001) according to the manufacturer’s instructions. DNAs from Kelly and SK-N-FI neuroblastoma cell lines were used as controls. Signals were detected by using a BIORAD MP imaging System.

### *In vivo* growth inhibition analysis

To determine tumor growth inhibition by imetelstat, 6-thio-dG, etoposide, and drug combinations, 5 × 10^6^ to 1 × 10^7^ neuroblastoma cells were resuspended in 200 µl Matrigel (Thermofisher Scientific, Waltham, USA) and injected subcutaneously into the flank of immunodeficient athymic nude mice (Charles River Laboratories, Sulzfeld, Germany). Tumor volumes were measured in (LxWxD)/2 mm^3^ every two days by digital caliper, and animal weights were tracked daily. When tumors reached > 80 mm^3^ average volume, the mice were randomly divided into control and treatment groups (12 animals per group). Intraperitoneal injections of imetelstat (30 mg/kg in aqua ad inj.), or vehicle alone were administered twice weekly. Etoposide (15 mg/kg) and/or 6-thio-dG (2.5 mg/kg), or control (DMSO 5%) were injected three times/week intraperitoneally. Mice were euthanized by cervical dislocation at a tumor volume > 1000 mm^3^. All animal experiments were performed according to German ethics guidelines and law (file number: 84–02.04.2016.A064 and 81–02.04.2017.A508) meeting the ARRIVE guidelines.

### TUNEL assay

Fresh frozen tumors were cut onto glass slides at 6 µm after which a terminal deoxynucleotidyl transferase mediated dUTP nick end labeling (TUNEL) assay was used to determine the rate of apoptotic cells. TUNEL assay was performed using an In Situ Cell Death Detection Kit, TMR red (Roche) according to the manufacturer’s instructions. Samples were mounted with VectaShield mounting media containing 4′,6-diamidino-2-phenylindole dihydrochloride (Vector Laboratories). Images were acquired using a Leica DM 5550B fluorescent light microscope system and full z-stacks were taken at 0.5 µm and projected using CytoVision® 7.6 software (Leica).

### Statistical analyses

The data obtained are presented as mean values ± standard deviation (SD). Comparisons of different groups for statistical significance were analyzed using a two-tailed, unpaired Student t test. A *p*-value < 0.05 was considered significant. Statistical analyses were performed using the python packages pandas and scipy, while visualization was performed using matplotlib, seaborn and lifelines distributed through pypi.org under python version 3.8.10. Non-parametric variables between groups were compared using the Mann–Whitney-U test. Statistical significance of Kaplan Meier estimates were calculated using logrank test.

## Results

### 6-thio-dG has synergistic anti-tumor effects with cytotoxic drugs in neuroblastoma cell lines *in vitro*

To evaluate potential synergistic effects of the telomerase substrate nucleoside precursor 6-thio-dG with other compounds that are active in neuroblastoma (the genotoxic drugs etoposide and doxorubicin, and the *ALK* inhibitor ceritinib), we assessed the effect of these compounds on cell viability individually and in combinations in eight neuroblastoma cell lines *in vitro* (CLB-GA, BE(2)-C, SH-SY5Y, KELLY, GI-MEN, TR-14, IMR-32 and LS). All eight cell lines harbor telomerase activity, however, they differ in their genomic status of *TERT*, *MYCN* and *ALK* (Suppl. Table 1). We found that all tested compounds impaired cell viability in a dose-dependent manner in all cell lines (Supp. Fig. 1). Median growth inhibition to 50% (GI_50_) was 2.65 µM for 6-thio-dG, which is similar to previously reported GI_50_ concentrations in telomerase-positive neuroblastoma cell lines [4]. Median GI_50_ concentrations were 0.33 and 0.03 µM for etoposide and doxorubicin, respectively. Exposure to the *ALK* inhibitor ceritinib resulted in more pronounced growth inhibition in *ALK*-mutated than in *ALK* wild-type cell lines (median GI_50_ 0.19 *versus* 0.61 µM, respectively).

**Fig. 1:**
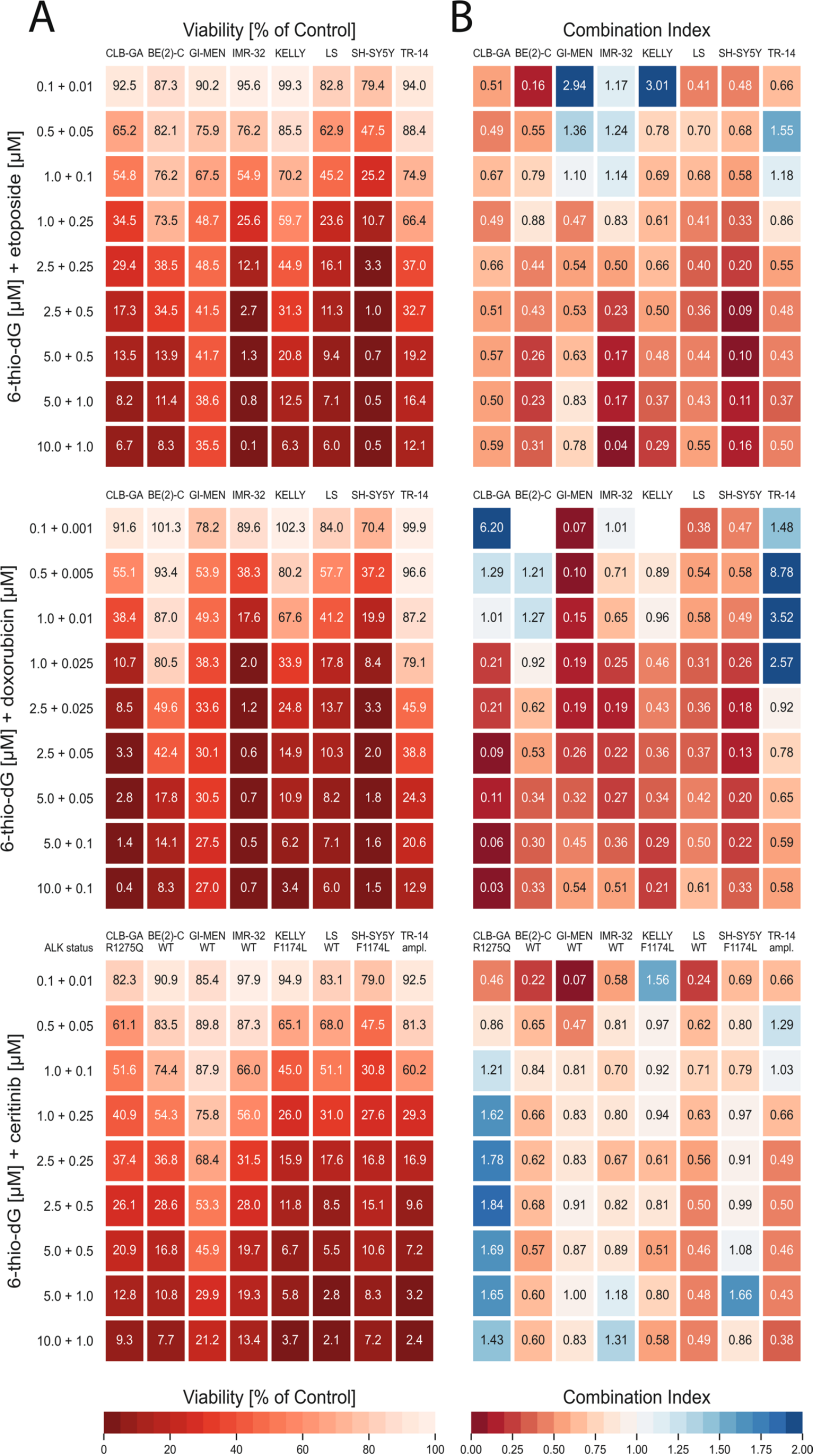
Drug synergy in neuroblastoma cell lines *in vitro*. Cell lines CLB-GA, GI-MEN, KELLY, BE(2)-C, IMR-32, LS, SH-SY5Y and TR-14 were treated at the indicated concentrations of drug combinations for 96 h. Genomic *ALK* status is indicated below cell line labels on top of bottom panels. **A** Cell viability of cell lines upon combination treatment, normalized to the response of the control (DMSO). **B** Combination Indices (CoIn) of cell lines upon combination treatment; red, synergy; blue, antagonism. Missing CoIn result from higher viability signals in the combination treatment as compared to the control signal

To assess potential synergy of 6-thio-dG treatment in combination with cytotoxic drugs or ceritinib, we exposed all cell lines with increasing concentrations of combinations of these compounds, which had been selected to overlap with the therapeutic ranges according to the individual GI_50_ of the single compounds (Fig. 1). As expected, cell viability decreased with increasing concentrations of compound combinations in all cell lines (Fig. 1A). We then calculated a combination index (CoIn) according to the Chou-Talalay method, and classified the effects based on the CoIn into strong synergy (CoIn < 0.3), synergy (CoIn = 0.3–0.7), moderate and weak synergy (CoIn = 0.7–0.9) and no synergy (CoIn > 0.9) [17]. Overall, we observed similar synergistic effects in all eight cell lines when treated with the combination 6-thio-dG plus etoposide and 6-thio-dG plus doxorubicin, with synergistic to strongly synergistic CoIns in the higher concentration ranges above the individual GI_50_ of the single compounds (Fig. 1B). Mean combination indices of the four highest concentrations (CoIn*) were 0.39 (95% confidence interval [CI], 0.23–0.55) for 6-thio-dG plus etoposide, and 0.35 (95% CI, 0.21–0.50) for 6-thio-dG plus doxorubicin. In particular, strong synergies and close to zero percent viable cells were observed in cell lines IMR-32 and SH-SY5Y upon 6-thio-dG plus etoposide treatment, and in cell line CLB-GA upon 6-thio-dG plus doxorubicin. By contrast, weaker synergy was found for the combination of 6-thio-dG and the *ALK* inhibitor ceritinib in general (CoIn*, 0.87 [95% CI, 0.53–1.21]). In particular, we did not observe substantially different synergistic effects in *ALK*-mutated (CoIn*, 0.98 [95% CI, 0.12–1.83]) and in *ALK* wild-type (CoIn*_,_ 0.76 [95% CI, 0.35–1.17]) cell lines, with lowest CoIn* (1.65 [95% CI, 1.38–1.92]) in the *ALK*-mutated cell line CLB-GA, pointing towards antagonism in this case.

**Fig. 2:**
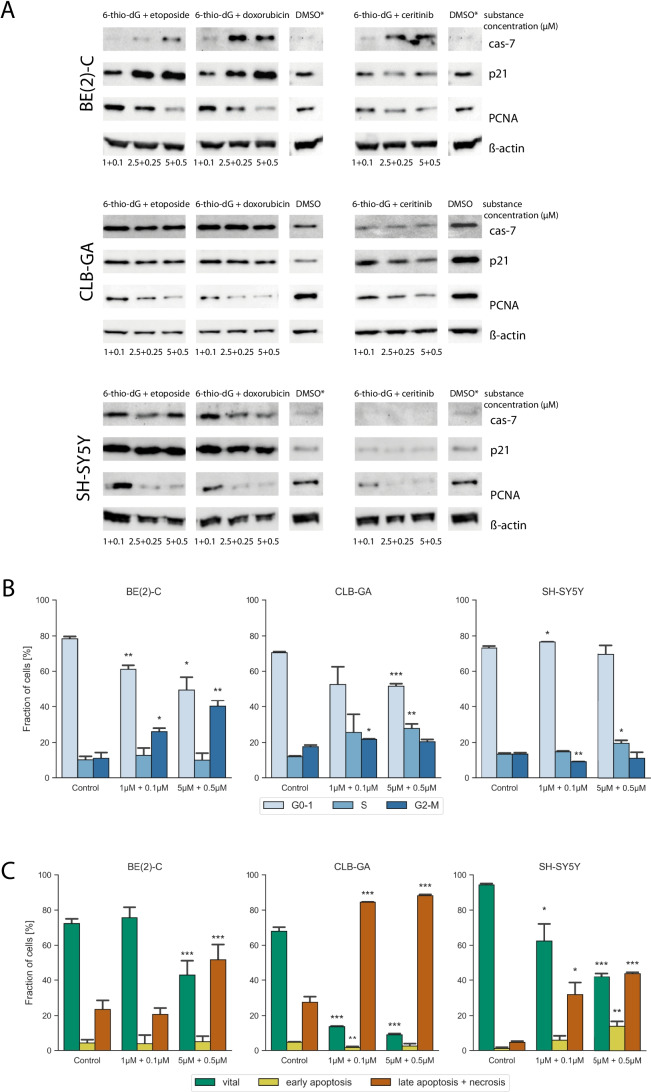
**A** Western blot analysis of cleaved caspase-7, p21^Waf1/Cip1^, and PCNA in cell-lines BE(2)-C, CLB-GA and SH-SY5Y after 48 h of the indicated treatment combinations or DMSO; ß-actin was used as loading control. Asterisks indicate identical DMSO markers on one membrane. **B** Cell cycle analysis as determined by flow cytometry, showing G0/G1- (light blue), S- (blue) and G2/M-phase (dark blue) in CLB-GA, SH-SY5Y and BE(2)-C after treatment with 6-thio-dG and etoposide or control for 48 h. **C** Annexin/PI flow cytometry analysis showing vital cells (green), early apoptosis (yellow) and late apoptosis with necrosis (orange) in CLB-GA, SH-SY5Y and BE(2)-C after a treatment with 6-thio-dG and etoposide or control for 48 h. *P*-Values are indicated by asterisks; (*) *p* < 0.05, (**) *p* < 0.01, (***) p < 0.001

For further analyses, we selected neuroblastoma cell lines that represent three distinct genetic groups, i. e., cells bearing a *TERT* rearrangement (CLB-GA), *MYCN* amplification (BE(2)-C), or elevated *TERT* expression without such genomic alterations (SH-SY5Y). To assess which mechanisms may contribute to cell growth inhibition upon combination treatment, we first determined changes of cleaved caspase-7 as a marker of apoptosis, p21^Waf1/Cip1^ as a marker of cell cycle arrest, and PCNA as a marker of cellular proliferation by immunoblotting in cell lines SH-SY5Y, CLB-GA and BE(2)-C (Fig. 2A). We observed concentration-dependent decreases in PCNA levels after exposure to all three combinations (i.e., 6-thio-dG plus etoposide, 6-thio-dG plus doxorubicin, and 6-thio-dG plus ceritinib) in all cell lines, pointing towards impaired proliferation upon treatment. Decrease of PCNA was accompanied by induction of p21^Waf1/Cip1^ in cells exposed to 6-thio-dG plus etoposide and 6-thio-dG plus doxorubicin, but not 6-thio-dG plus ceritinib, indicating that impaired proliferation goes along with cell cycle arrest in the two combinations containing cytotoxic drugs, but not in the combination of 6-thio-dG and *ALK* inhibitor. In line with these results, we found an increase of the G2/M fraction in BE(2)-C cells (Fig. 2B) and a decrease of phosphorylated CDK1 in CLB-GA and SH-SY5Y cells upon treatment with 6-thio-dG plus etoposide (Suppl. Fig. 2), pointing towards cell cycle arrest in G2, which has been reported previously for cells treated with etoposide [19]. We also observed a dose-dependent increase of cleaved caspase-7 after exposure of all three combinations in cell line BE(2)-C, whereas maximum levels were already achieved at the lowest concentration of combinations with etoposide and doxorubicin in cell lines SH-SY5Y and CLB-GA (Fig. 2A). By contrast, no induction of cleaved caspase-7 was detected in these two cell lines, both of which harbor an oncogenic *ALK* mutation, upon treatment with 6-thio-dG plus ceritinib. Moreover, we observed a significant increase of late apoptotic cells in CLB-GA, SH-SY5Y and BE(2)C treated with 6-thio-dG and etoposide using flow cytometry with annexin V/PI staining (Fig. 2C), and an increase of cleaved-PARP in all three cell lines in a concentration-dependent manner (Suppl. Fig. 2), supporting the notion of caspase-mediated apoptosis following 6-thio-dG and etoposide treatment.

These data suggest that decreased cell viability caused by 6-thio-dG in combination with cytotoxic drugs is at least partly due to apoptosis, while this effect may vary after exposure to 6-thio-dG in combination with ceritinib. Together, our results indicate that combination treatment of 6-thio-dG plus genotoxic drugs, but not 6-thio-dG plus *ALK* inhibition, leads to growth inhibition by inducing both cell cycle arrest and apoptosis.

### A combination of 6-thio-dG and etoposide can improve tumor growth inhibition and survival in neuroblastoma xenografts

**Fig. 3:**
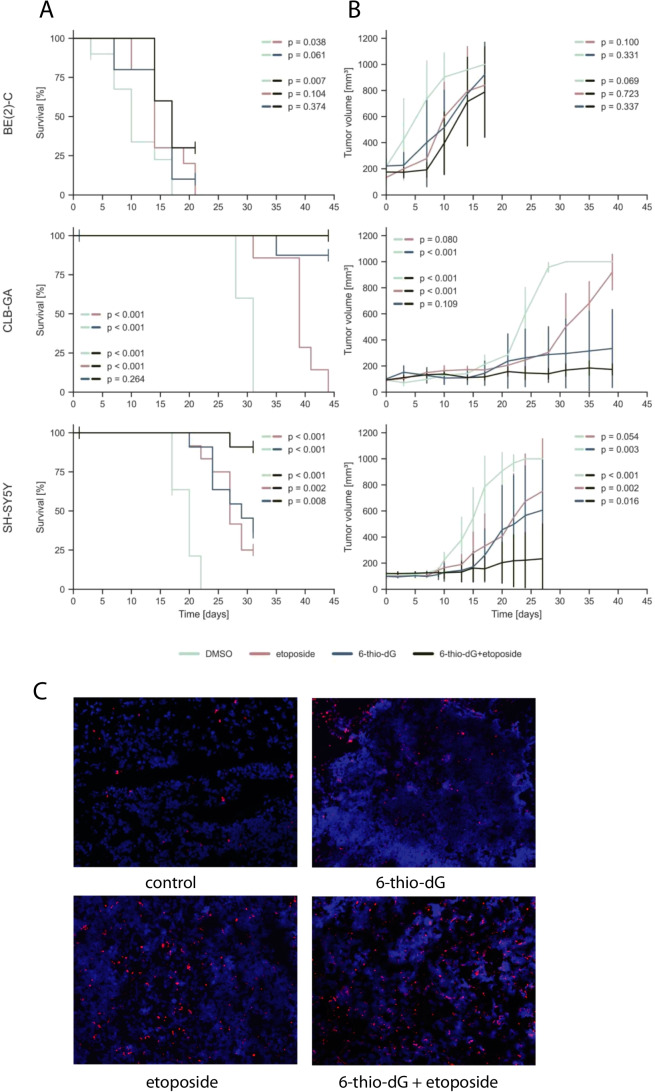
**A** Kaplan–Meier survival estimates for survival of athymic nude mice bearing xenografts of human neuroblastoma cell lines CLB-GA, SH-SY5Y and BE(2)-C upon intraperitoneal administration of 6-thio-dG (blue), etoposide (red), DMSO (mint) or combination of 6-thio-dG and etoposide (black) every other day; each group, *n* = 12. **B** Tumor growth of neuroblastoma xenografts as indicated in panel (A). *P*-values were calculated for the last day of measurement between two treatment options. **C** TMR red TUNEL signals indicating apoptosis in CLB-GA xenograft tumors treated with 6-thio-dG, etoposide, 6-thio-dG plus etoposide, or vehicle (DMSO). Cell nuclei were counterstained with DAPI

Based on the *in vitro* observations, we expected synergistic growth inhibitory effects *in vivo* for the combination of 6-thio-dG and genotoxic drugs. As we found comparable CoIn for both combination treatments (6-thio-dG/etoposide and 6-thio-dG/doxorubicin) in BE(2)-C and in SH-SY5Y, we opted to test a combination of 6-thio-dG (2 mg/kg) and etoposide (15 mg/kg) in mouse xenografts of these two cell lines. In addition, we selected CLB-GA, representing a cell line that bears a *TERT* rearrangement. We found that combination treatment was well tolerated, with no effects on mouse weight, appearance or behavior (Supp. Fig. 3). Telomer length was not significantly altered in xenograft tumors after 28 days of treatment with 6-thio-dG and etoposide *versus* vehicle alone (Suppl. Fig. 4). However, we observed significantly improved survival and attenuated tumor growth in cell lines CLB-GA and SH-SY5Y when compared to mono-therapy with etoposide, and in cell line SH-SY5Y when compared to mono-therapy with 6-thio-dG (Fig. 3A and B). The latter treatment was already highly efficacious in CLB-GA xenografts, however, combination treatment almost completely abolished tumor growth in this model. In line with these findings, we observed an increase of apoptotic cells in CLB-GA tumors treated by 6-thio-dG/etoposide combination, in particular when compared to control or 6-thio-dG mono-therapy (Fig. 3C). By contrast, combination treatment of BE(2)-C xenograft models did not improve tumor growth inhibition and survival over etoposide or 6-thio-dG mono-therapy.

### Imetelstat impairs telomerase activity and tumor growth in neuroblastoma

**Fig. 4:**
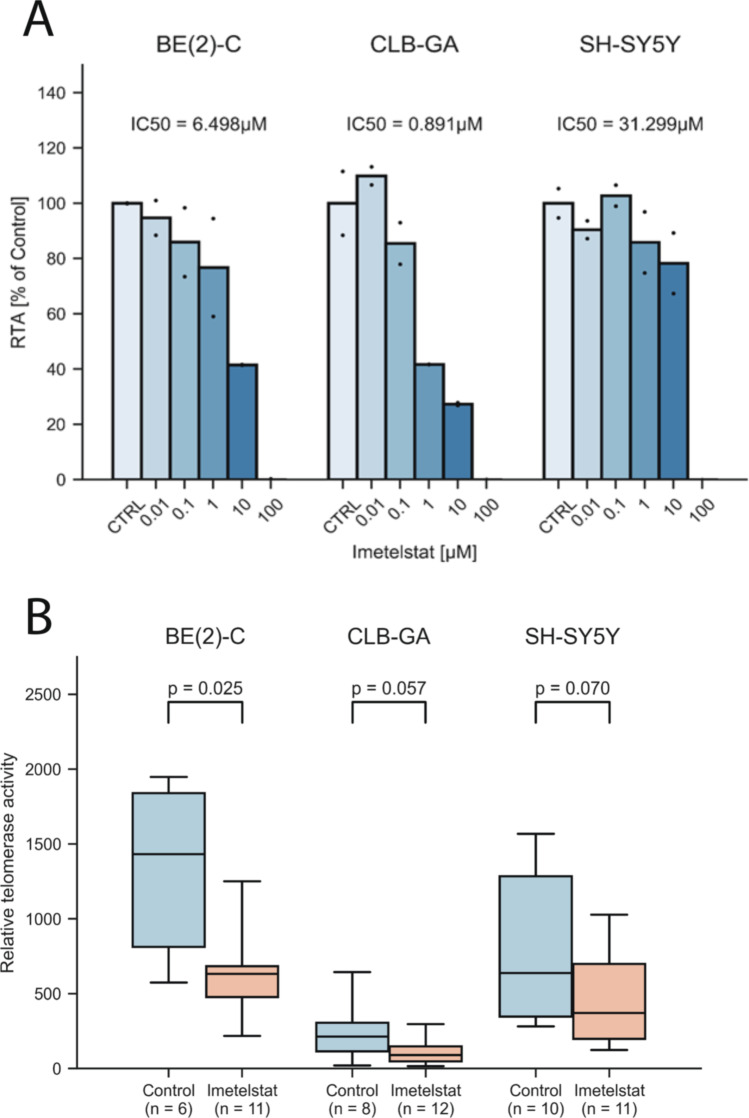
**A** Telomerase activity in neuroblastoma cell lines *in vitro* after 24 h incubation with imetelstat. Dots represent individual values. **B** Telomerase activity in neuroblastoma xenografts *in vivo* after intraperitoneal treatment with imetelstat (30 mg/kg) every other day for 17–42 days until tumors reached 1000 mm^3^; control animals received vehicle only (H_2_O). Bars represent standard deviation

**Fig. 5:**
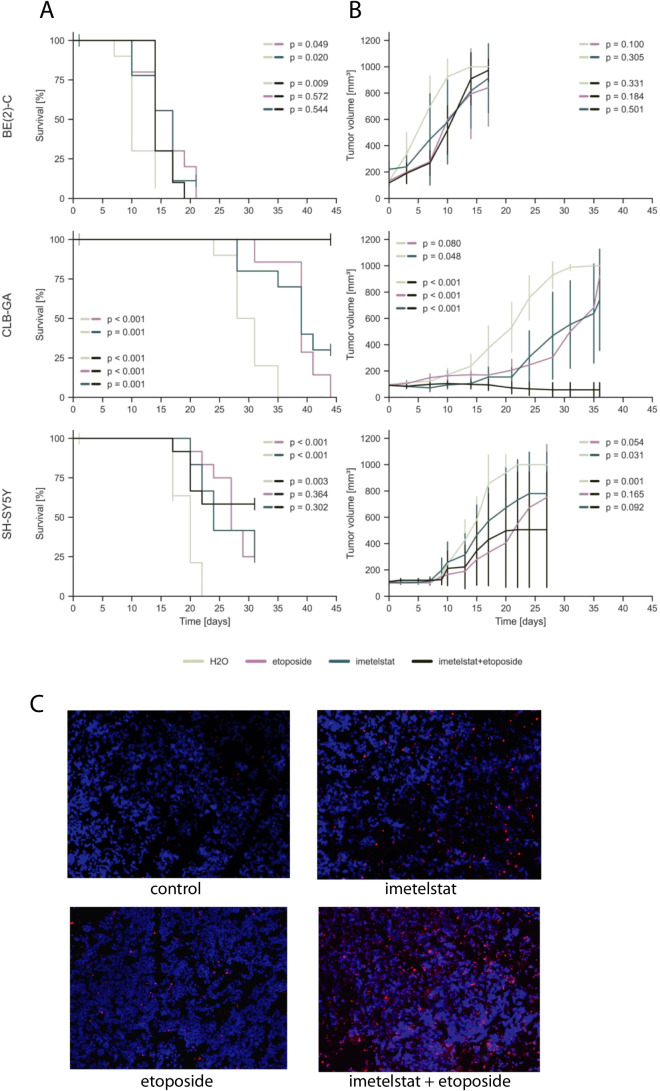
**A** Kaplan–Meier estimates for survival of athymic nude mice bearing xenografts of human neuroblastoma cell lines CLB-GA, SH-SY5Y, and BE(2)-C upon intraperitoneal administration of imetelstat (blue), etoposide (purple), H_2_O (grey) or combination of imetelstat and etoposide (black) every other day; each group, *n* = 10. **B** Tumor growth of neuroblastoma xenografts as indicated in panel (A). P-values were calculated for the last day of measurement between two treatment options. **C** TMR red TUNEL signals indicating apoptosis in CLB-GA xenograft tumors treated with imetelstat, etoposide, imetelstat plus etoposide, or vehicle (H_2_O). Cell nuclei were counterstained with DAPI

We next aimed to assess whether direct inhibition of telomerase by imetelstat has similar effects on neuroblastoma growth as induction of telomere dysfunction by 6-thio-dG. First, we determined the effect of imetelstat on telomerase activity in the human neuroblastoma cell lines CLB-GA, BE(2)-C and SH-SY5Y *in vitro*. We found that imetelstat impaired telomerase activity in the telomerase-rearranged cell line CLB-GA (IC_50_ = 0.89 µM) and the *MYCN*-amplified cell line BE(2)-C (IC_50_ = 6.5 µM) at substantially lower concentrations than in SH-SY5Y cells that express high levels of *TERT* without such alterations (IC_50_ = 31.3 µM; Fig. 4A). We next treated mice bearing subcutaneous xenografts of these three cell lines with imetelstat (30 mg/kg twice a week), and compared telomerase activity in treated xenograft tumors with that of controls. We found that, overall, telomerase activity decreased by roughly 50%, which was a significant reduction in BE(2)-C xenografts and a trend in CLB-GA and SH-SY5Y xenografts (Fig. 4B). We also noted that imetelstat treatment significantly improved survival of mice bearing xenografts in all three cell lines, while a significant effect on xenograft growth was observed for CLB-GA and SH-SY5Y, but not for BE(2)-C tumors (Fig. 5A and B). By contrast, imetelstat treatment did not affect tumor growth or survival in xenograft models of neuroblastoma cell line SK-N-FI, in which telomere maintenance is conferred by ALT (Suppl. Fig. 5). Together, these data suggest that direct inhibition of telomerase by imetelstat improves survival of mice bearing neuroblastoma xenografts, although inhibition of telomerase activity in these tumors is incomplete**.**

### A combination of imetelstat and etoposide can improve tumor growth inhibition and survival in neuroblastoma xenografts

We next aimed to determine whether a combination of imetelstat and etoposide may act synergistically in neuroblastoma xenografts, analogous to 6-thio-dG. Similar to 6-thio-dG combination therapy, we noted that treatment with imetelstat in combination with etoposide was well tolerated by the animals (Supp. Fig. 3). We found that treatment with this combination significantly improved mouse survival in all three models over treatment with control (Fig. 5A). Similar to combination treatment of 6-thio-dG with etoposide, we observed a significant improvement of survival in CLB-GA xenograft mice upon combination treatment when compared to imetelstat or etoposide mono-therapy, which was due to almost complete inhibition of tumor growth by combination treatment (Fig. 5A). We also found an increase of apoptotic cells in tumors treated by imetelstat/etoposide combination, in particular when compared to control and to imetelstat mono-therapy (Fig. 5C). By contrast, survival of mice and tumor growth was not significantly altered in the two other models in comparison to imetelstat or etoposide monotherapy.

## Discussion

We here demonstrate that combinations of telomerase interacting compounds and cytotoxic drugs synergistically inhibit tumor growth in preclinical neuroblastoma models, thus offering a promising therapeutic approach for patients with telomerase-positive high-risk neuroblastoma. By contrast, we did not observe significant synergistic effects of 6-thio-dG in combination with the *ALK* inhibitor ceritinib, both in *ALK*-mutated and *ALK*-wildtype neuroblastoma cells*.*

Induction of *TERT* occurs in the majority of high-risk neuroblastomas, and telomerase has thus been considered as a promising tractable target [2, 5, 6]. We previously have shown that treatment with the telomerase substrate nucleoside precursor 6-thio-dG attenuates tumor growth of telomerase-positive neuroblastoma cell lines, both *in vitro* and *in vivo*, but not that of ALT-positive tumors [4]. Therapy with 6-thio-dG results in telomere dysfunction without substantial changes of telomere lengths [10]. In line with this mechanism, telomere lengths of cells or tumors treated with 6-thio-dG and etoposide did not differ from controls (Suppl. Fig. 4). Another recent study demonstrated anticancer effects of the BET bromodomain inhibitor OTX015 and the proteasome inhibitor carfilzomib by *TERT* down-regulation in *TERT*-rearranged neuroblastoma cell lines [20]. Similarly, we here demonstrate that direct inhibition of telomerase by imetelstat, which competitively inhibits telomerase enzymatic activity through binding to the template region of the RNA component of telomerase [21], leads to attenuation of neuroblastoma growth. Imetelstat treatment reduced telomerase activity by roughly 50% in mouse xenograft tumors, which highlights the growth-inhibitory effect of even incomplete telomerase inhibition in telomerase-dependent tumors. These data suggest that enhancing the *in vivo* inhibitory efficiency of direct telomerase inhibitors may further increase their anticancer effects in telomerase-positive neuroblastoma.

While previous studies substantiated telomerase as an actionable target in neuroblastoma, it had remained unclear how such treatment strategies could be combined with current therapy regimens for high-risk neuroblastoma patients. We here demonstrate that both 6-thio-dG and imetelstat act synergistically with etoposide, a cytotoxic drug frequently used in contemporary neuroblastoma treatment regimens [22], in attenuating tumor growth, at least in a fraction of neuroblastoma. We found, however, that the response of the *MYCN*-amplified and p53-mutated cell line BE(2)-C to combination treatment *in vitro* was relatively poor, and that no synergistic effects were found in BE(2)-C xenografts. While the mechanistic understanding of this observation remains elusive, it has to be noted that this cell line had been generated from a chemotherapy refractory neuroblastoma [23], suggesting that DNA damaging agents may be largely ineffective in this case. This notion is supported by the fact that BE(2)-C showed the highest *in vitro* GI_50_ for etoposide in all tested cell lines, which was up to ten-fold higher than those in CLB-GA and SH-SY5Y cells (Suppl. Fig. 1), pointing towards etoposide resistance of BE(2)-C cells.

At the molecular level, we found that combination treatment of 6-thio-dG and cytotoxic drugs led to robust induction of cleaved caspase-7, cleaved PARP and p21^Waf1/Cip1^, as well as DNA fragmentation. We also observed depletion of phosphorylated CDK1 in a concentration dependent manner in two cell lines, pointing towards an increase of cells in G2/M arrest. These findings suggests that the anticancer effects of combination treatment were due to both apoptosis and cell cycle arrest, as described previously for 6-thio-dG mono-therapy [4, 10, 24]. By contrast, we did not observe robust synergistic effects of 6-thio-dG with the *ALK* inhibitor ceritinib, and this combination failed to induce cleaved caspase-7 and p21^Waf1/Cip1^ in both of the *ALK* mutated cell lines examined. While the reason for the lack of synergy between the telomere dysfunction-inducing compound 6-thio-dG and the *ALK* inhibitor ceritinib remains to be addressed, one may speculate that this finding is due to a preponderance of cytostatic over cytotoxic effects of the *ALK* inhibitor [8, 25–28], which may impair incorporation of 6-thio-dG into telomeres and subsequent telomere dysfunction-induced cell death.

Targeted therapies have been integrated in cancer therapy based on suspected synergistic effects of molecular functions of the targets or empiric evidence of additive or synergistic effects in cell line and animal models, some of which led to an improved clinical outcome of patients [29, 30]. In cases with hyperactivated *RAS-MAPK* signaling, combination therapy is already being tested clinically in relapsed high-risk neuroblastoma patients [31]. Our study provides a rationale for combining telomerase-interacting compounds and genotoxic drugs in patients with telomerase-positive neuroblastoma. However, even when a drug combination exhibits additivity or synergy in pre-clinical models, patient-to-patient variability and cross resistance can make independent action the dominant mechanism in clinical populations [32]. Therefore, each combination has to be carefully examined in prospective clinical trials.

## Conclusion

Our data indicate that telomerase inhibition acts synergistically with genotoxic drugs in telomerase-positive neuroblastoma models and may represent a promising treatment approach to be evaluated in high-risk neuroblastoma patients.

## Supplementary Information

Below is the link to the electronic supplementary material.

Supplementary file1 (PDF 222 KB)

Supplementary file2 (JPG 1700 KB) Suppl. Fig. 1: Relative cell viability determined by CellTiter-Glo assay in neuroblastoma cell lines treated with various concentrations of 6-thio-dG, etoposide, doxorubicin, or ceritinib. Error bars indicate standard deviation. Half maximal inhibitory concentrations (GI_50_) of individual compounds are given for each cell line

Supplementary file3 (PDF 426 KB) Suppl. Fig. 2: Western blot analysis of PARP, cleaved-PARP, and pCDK1 in cell-lines BE(2)-C, CLB-GA and SH-SY5Y after 48 hours of treatment with 6-thio-dG plus etoposide at the indicated concentrations or DMSO; ß-actin was used as loading control

Supplementary file4 (PDF 534 KB) Suppl. Fig. 3: Development of mouse weights (g) of the indicated treatment groups over time. Error bars indicate standard deviation

Supplementary file5 (PDF 435 KB) Suppl. Fig. 4: Mean telomere lengths (kb) of SH-SY5Y cells as determined by telomere restriction fragment assay after treatment with control or 6-thio-dG and etoposide for 96 h *in vitro*, and after 4-week-treatment of xenograft tumors

Supplementary file6 (JPG 31 KB) Suppl. Fig. 5: (A) KaplanMeier estimates for survival of athymic nude mice bearing xenografts of human neuroblastoma cell line SK-N-FI treated with imetelstat versus vehicle alone. (B) Tumor growth of SK-N-FI xenografts as indicated in (A). P-values were calculated for the last day of measurement

## Data Availability

The datasets generated and analyzed during the current study are not publicly available but are available from the corresponding author upon reasonable request.

## Abbreviations

*6-thio-dG*: 6-Thio-2’-deoxyguanosine
*ALK*: Anaplastic lymphoma kinase
*ALT*: Alternative lengthening of telomeres
*ATRX*: Transcription regulator
*CO2*: Carbon dioxide
*CoIn*: Combination index
*CI*: Confidence intervall
*DMSO*: Dimethyl sulfoxide
*DNA*: Desoxyribonucleic acid
*FCS*: Fetal calf serum
*GI50*: 50% Growth inhibition
*IC*_*50*_: 50% Inhibitory concentration
*hTERC*: Human telomerase C complex
*MYCN*: Term of the proto-oncogen
*NB*: Neuroblastoma
*RAS-MAPK*: Term of the signaling pathway
*RNA*: Ribonucleic acid
*TERT*: Telomerase reverse transcriptase
*TRAP*: Telomeric repeat assay protocol
*TRF*: Telomere restriction fragment
*TUNEL*: Terminal deoxynucleotidyl transferase mediated dUTP nick end labeling
