# Supplementary material for: Telomerase-targeting compounds Imetelstat and 6-thio-dG act synergistically with chemotherapy in high-risk neuroblastoma models
Source: Cell Oncol (Dordr). 2022 Aug 12;45(5):991–1003. doi: 10.1007/s13402-022-00702-8 (PMC9579108; doi:10.1007/s13402-022-00702-8)
Supplement: Supplementary file 1 — Supplementary file1 (PDF 222 KB) [file 13402_2022_702_MOESM1_ESM.pdf]

Suppl. Table 1

**Cell line**	**TMM**	***MYCN/TERT* status**	***TERT* expression**	***ALK* status**
**BE(2)C**	Telomerase pos.	*MYCN* amplified	10.31	WT
**CLB-GA**	Telomerase pos.	*TERT* rearr.	13.67	R1275Q
**GI-MEN**	Telomerase pos.	*TERT* rearr.	13.88	WT
**IMR-32**	Telomerase pos.	*MYCN* amplified	10.95	WT
**KELLY**	Telomerase pos.	*MYCN* amplified	11.37	F1174L
**LS**	Telomerase pos.	*MYCN* amplified	10.49	WT
**SH-SY5Y**	Telomerase pos.	WT	12.38	F1174L
**SK-NFI**	ALT	WT	8.81	D1529E
**TR-14**	Telomerase pos.	*MYCN* amplified	8.92	amplified

Suppl. Table 1: Molecular characteristics of cell lines used in this study; TMM, telomere maintenance mechanism; WT, wild-type; ALT, alternative lengthening of telomeres. *TERT* expression is given as $\log_2$ intensity according to microarray analysis.
