# Supplementary figures and images for: Telomerase-targeting compounds Imetelstat and 6-thio-dG act synergistically with chemotherapy in high-risk neuroblastoma models
Source: Cell Oncol (Dordr). 2022 Aug 12;45(5):991–1003. doi: 10.1007/s13402-022-00702-8 (PMC9579108; doi:10.1007/s13402-022-00702-8)

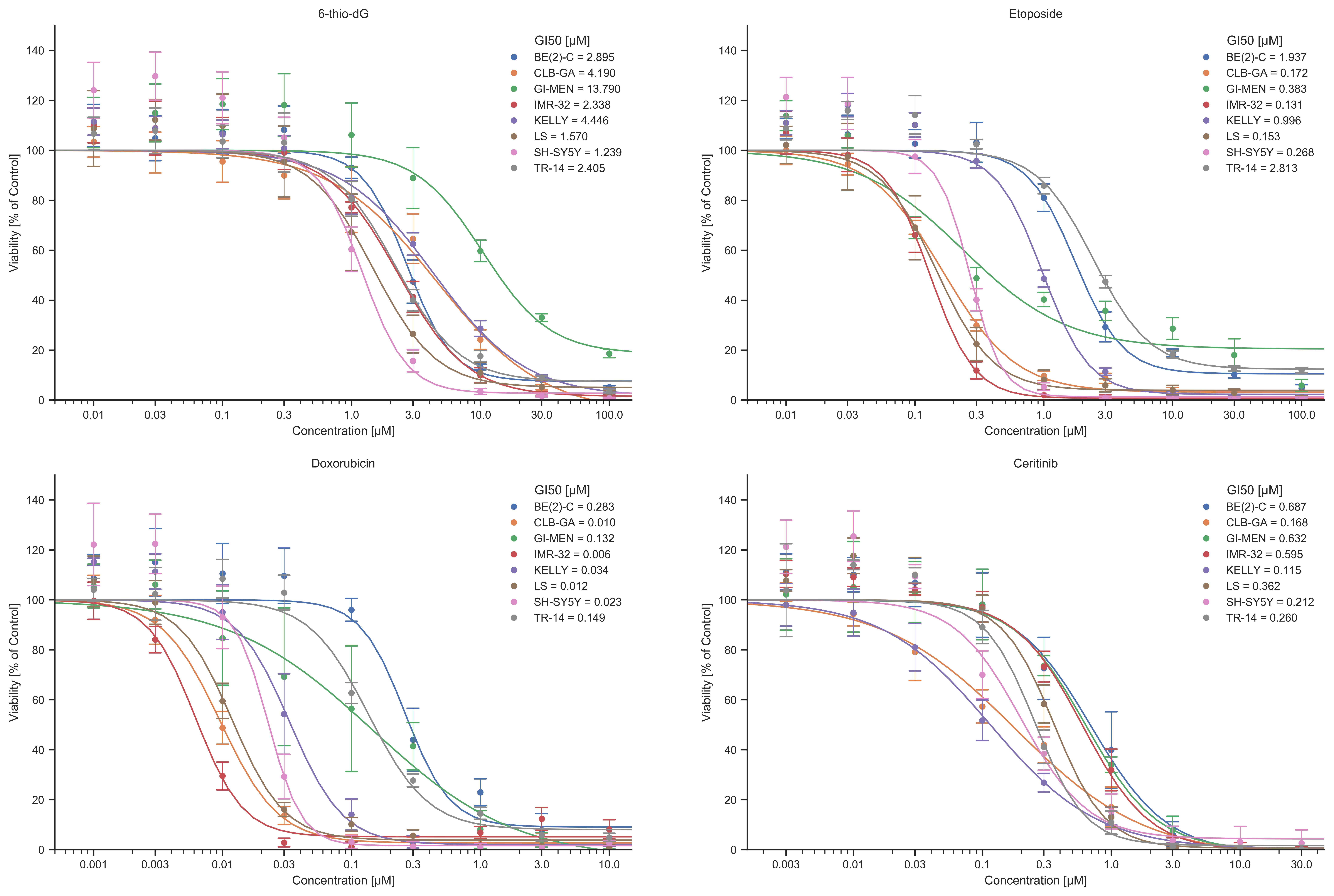

Supplement: Supplementary file 2 — Supplementary file2 (JPG 1700 KB) Suppl. Fig. 1: Relative cell viability determined by CellTiter-Glo assay in neuroblastoma cell lines treated with various concentrations of 6-thio-dG, etoposide, doxorubicin, or ceritinib. Error bars indicate standard deviation. Half maximal inhibitory concentrations (GI50) of individual compounds are given for each cell line [file 13402_2022_702_MOESM2_ESM.jpg]

Suppl. Fig. 2

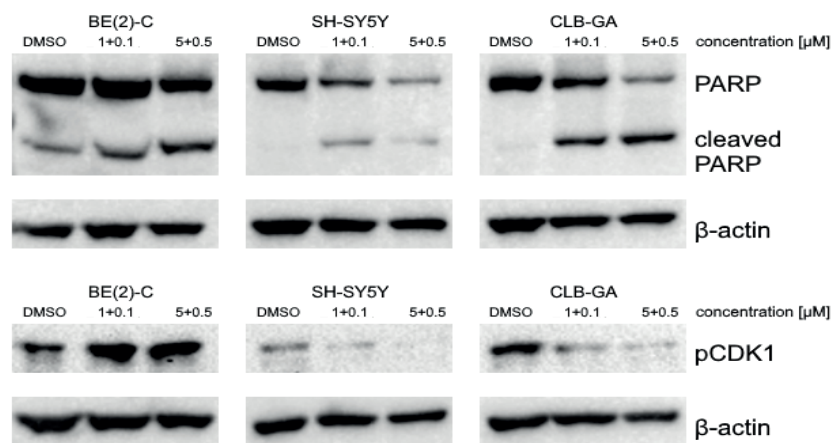

Supplement: Supplementary file 3 — Supplementary file3 (PDF 426 KB) Suppl. Fig. 2: Western blot analysis of PARP, cleaved-PARP, and pCDK1 in cell-lines BE(2)-C, CLB-GA and SH-SY5Y after 48 hours of treatment with 6-thio-dG plus etoposide at the indicated concentrations or DMSO; ß-actin was used as loading control [file 13402_2022_702_MOESM3_ESM.pdf]

Suppl. Fig. 3

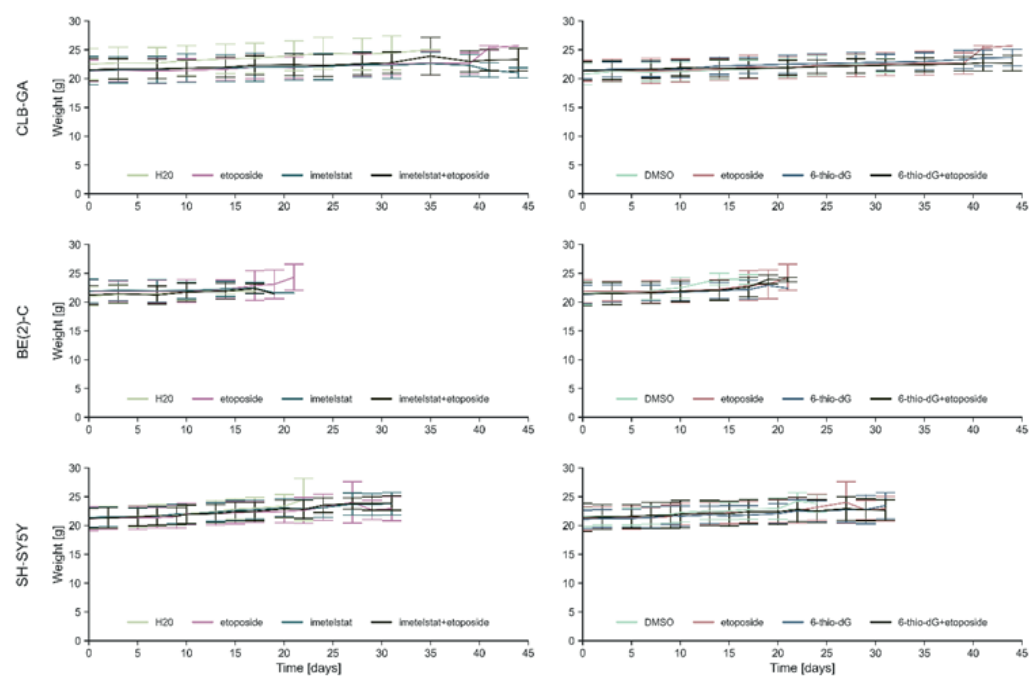

Supplement: Supplementary file 4 — Supplementary file4 (PDF 534 KB) Suppl. Fig. 3: Development of mouse weights (g) of the indicated treatment groups over time. Error bars indicate standard deviation [file 13402_2022_702_MOESM4_ESM.pdf]

Suppl. Fig. 4

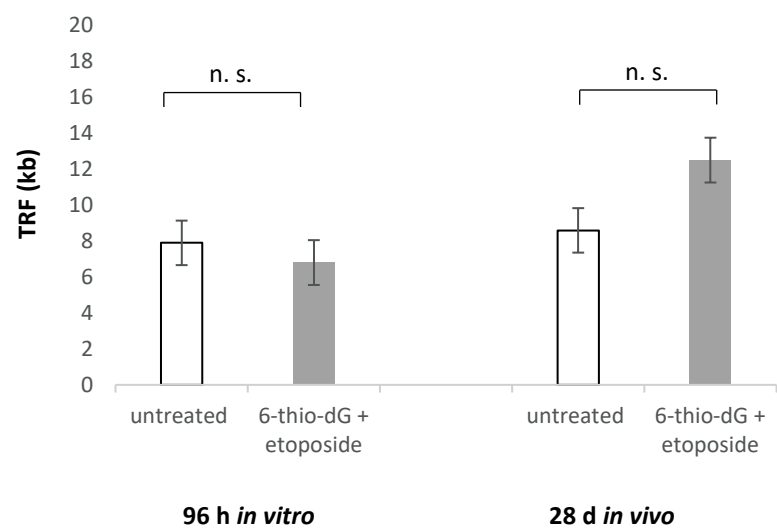

Supplement: Supplementary file 5 — Supplementary file5 (PDF 435 KB) Suppl. Fig. 4: Mean telomere lengths (kb) of SH-SY5Y cells as determined by telomere restriction fragment assay after treatment with control or 6-thio-dG and etoposide for 96 h in vitro, and after 4-week-treatment of xenograft tumors [file 13402_2022_702_MOESM5_ESM.pdf]

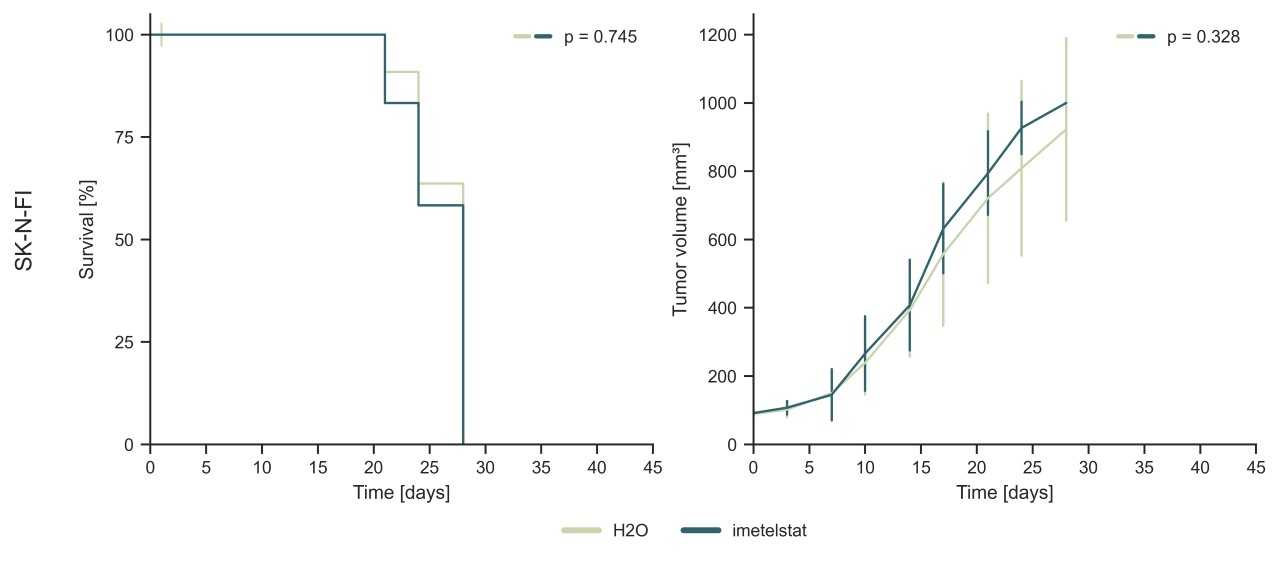

Supplement: Supplementary file 6 — Supplementary file6 (JPG 31 KB) Suppl. Fig. 5: (A) KaplanMeier estimates for survival of athymic nude mice bearing xenografts of human neuroblastoma cell line SK-N-FI treated with imetelstat versus vehicle alone. (B) Tumor growth of SK-N-FI xenografts as indicated in (A). P-values were calculated for the last day of measurement [file 13402_2022_702_MOESM6_ESM.jpg]
